# Supplementary material for: A cross-sectional study for the mental health status and sleep quality among college students in Macao during the COVID-19 pandemic
Source: PeerJ. 2021 Dec 7;9:e12520. doi: 10.7717/peerj.12520 (PMC8663623; doi:10.7717/peerj.12520)
Supplement: Supplemental Information 2 [file peerj-09-12520-s002.pdf]

问卷编号：□□-□□□□□□□□□　　**问卷录入1：______________________**

问卷审核：______________________　　**问卷录入2：______________________**

新冠肺炎疫情影响下大学生心理健康调查问卷

第一部分 基本资料

1.你的出生年月：_______年_______月

2.你的性别：○女　○男

第二部分 相关行为

1.你在疫情前是否有吸烟的习惯：○是　○否（跳转至5）

2.你在疫情前每日平均吸烟支数：○1~5支　○6~10支　○11~20支　○20支以上

3.你在疫情期间平均每日吸烟支数是否有增加：○是　○否（跳转至7）

4.增加吸烟支数的原因是：________________________________（跳转至7）

5.你在疫情期间是否有尝试吸烟的行为：○是　○否（跳转至7）

6.你尝试吸烟的原因是：________________________________

7.你在疫情前是否有饮酒的习惯：○是　○否（跳转至10）

8.你在疫情期间饮酒次数和饮酒量是否有增加：○是　○否（跳转至12）

9.增加饮酒次数和饮酒量的原因是：________________________________

10.你在疫情期间是否有尝试饮酒的行为：○是　○否（跳转至12）

11.你尝试饮酒的原因是：________________________________

12.在疫情期间你是否遭受到网络骚扰或网络暴力：○是　○否

13.在疫情期间你是否遭受到家庭暴力（包括家庭冷暴力）：○是　○否

14.在疫情期间你是否<u>自行</u>通过药物缓解包括失眠多梦、神经性头痛、食欲不振、神经性腹泻等症状

或者通过使用药物追求神经兴奋：○是　○否

15.在疫情期间你是否寻求过或尝试寻求心理疏导：○是　○否

第三部分 12项一般健康问卷（GHQ-12）

请你回忆并选择在疫情爆发（以武汉宣布“封城”日即1月23日为界点）后的1个月内，最符合你身体情况表现的选项：

1.在做事情的时候，能集中精神吗？	○能集中	○和平时一样	○不能集中	○完全不能
2.有由于过分担心而失眠的情况吗？	○没有过	○和平时一样	○有过	○总这样
3.觉得自己是有用的人吗？	○有用	○和平时一样	○没有用	○完全没有用
4.觉得自己有决断力吗？	○有	○和平时一样	○没有	○完全没有
5.总是处于紧张状态吗？	○不紧张	○和平时一样	○紧张	○非常紧张
6.觉得自己不能解决问题吗？	○能	○和平时一样	○不能	○完全不能
7.能享受日常活动吗？	○能	○和平时一样	○不能	○完全不能
8.能够面对你所面临的问题吗？	○能	○和平时一样	○不能	○完全不能
9.感到痛苦、忧虑吗？	○不觉得	○和平时一样	○觉得	○总是觉得
10.失去自信了吗？	○没有	○和平时一样	○失去	○完全失去
11.觉得自己是没有价值的人吗？	○没有	○和平时一样	○觉得	○总是觉得
12.觉得所有的事情都顺利吗？	○顺利	○和平时一样	○不顺利	○完全不顺利

第四部分 匹兹堡睡眠质量指数量表（PSQI）

请你回忆并选择在疫情爆发（以武汉宣布“封城”日即1月23日为界点）后的1个月内你的睡眠质量情况：

（提示：这些问题是关于你的实际睡眠状况的调查，这仅仅与你的睡眠习惯有关。请选择或填写最符合你白天和晚上睡眠实际情况的选项，并尽可能地做精确回答。划有横杠的部分是需要自己填写。）

1.在一个月中，你晚上上床睡觉通常是______:______（填写具体时刻）

2.在一个月中，你每晚通常要多长时间才能入睡（从上床到入睡）：_________分钟

3.在一个月中，你每天早上通常______:______（填写具体时刻）点钟起床

4.在一个月中，你每晚实际睡眠的时间为_________小时（注意不等同于卧床时间）

5.在一个月中，你是否因下列情况影响睡眠而烦恼，选择一最符合你的选项作为答案，并划“√”：

a.入睡困难（不能在30分钟内入睡） ① 无 ② ＜1次/周 ③ 1~2次/周 ④ ≥3次/周

b.夜间易醒或早醒 ① 无 ② ＜1次/周 ③ 1~2次/周 ④ ≥3次/周

c.夜间去厕所 ① 无 ② ＜1次/周 ③ 1~2次/周 ④ ≥3次/周

d.呼吸不畅 ① 无 ② ＜1次/周 ③ 1~2次/周 ④ ≥3次/周

e.咳嗽或鼾声高 ① 无 ② ＜1次/周 ③ 1~2次/周 ④ ≥3次/周

f.感觉冷 ① 无 ② ＜1次/周 ③ 1~2次/周 ④ ≥3次/周

g.感觉热 ① 无 ② ＜1次/周 ③ 1~2次/周 ④ ≥3次/周

h.做噩梦 ① 无 ② ＜1次/周 ③ 1~2次/周 ④ ≥3次/周

i.疼痛不适 ① 无 ② ＜1次/周 ③ 1~2次/周 ④ ≥3次/周

j.其他影响睡眠的事件：____________________________（如有，请填写，并勾选下方频率）

① 无 ② ＜1次/周 ③ 1~2次/周 ④ ≥3次/周

6.在一个月中，你认为自己的睡眠质量： ①很好 ② 较好 ③ 较差 ④ 很差

7.在一个月中，你用药物催眠的情况： ① 无 ② ＜1次/周 ③ 1~2次/周 ④ ≥3次/周

8.在一个月中，你感到困倦吗？ ① 无 ② ＜1次/周 ③ 1~2次/周 ④ ≥3次/周

9.在一个月中，你做事情的精力不足吗？ ①没有 ② 偶尔有 ③ 有时有 ④ 经常

问卷填答结束，再次感谢您的支持与参与，祝您和家人身体健康、学习和工作顺利！
